# Community mobility and participation assessment of manual wheelchair users: a review of current techniques and challenges

**DOI:** 10.3389/fnhum.2023.1331395

**Published:** 2024-01-05

**Authors:** Grace Fasipe, Maja Goršič, Mohammad Habibur Rahman, Jacob Rammer

**Affiliations:** ^1^Department of Biomedical Engineering, College of Engineering and Applied Science, University of Wisconsin-Milwaukee, Milwaukee, WI, United States; ^2^Department of Mechanical Engineering, College of Engineering and Applied Science, University of Wisconsin-Milwaukee, Milwaukee, WI, United States

**Keywords:** barriers, health-related quality of life, GPS tracking, community-based assessment, brain-computer interface, community mobility, community participation, wheelchair

## Abstract

According to the World Health Organization, hundreds of individuals commence wheelchair use daily, often due to an injury such as spinal cord injury or through a condition such as a stroke. However, manual wheelchair users typically experience reductions in individual community mobility and participation. In this review, articles from 2017 to 2023 were reviewed to identify means of measuring community mobility and participation of manual wheelchair users, factors that can impact these aspects, and current rehabilitation techniques for improving them. The selected articles document current best practices utilizing self-surveys, in-clinic assessments, and remote tracking through GPS and accelerometer data, which rehabilitation specialists can apply to track their patients’ community mobility and participation accurately. Furthermore, rehabilitation methods such as wheelchair training programs, brain-computer interface triggered functional electric stimulation therapy, and community-based rehabilitation programs show potential to improve the community mobility and participation of manual wheelchair users. Recommendations were made to highlight potential avenues for future research.

## 1 Introduction

Since the 1970s, data have been collected in the United States and worldwide, describing etiology of long-term manual wheelchair use by millions of individuals. As of 2015, there were over “2.7 million wheelchair users in the United States” (Koontz et al., 2015) (p1) alone. These numbers are attributed to conditions including but not limited to spinal cord injury (SCI), polio, stroke, cerebral palsy, myelomeningocele, and multiple sclerosis. Commencement of wheelchair use can drastically change an individual’s functional mobility and independence in daily living activities. While there is no guaranteed way to fully restore functionality for many conditions that result in wheelchair use, rehabilitation and training programs can help reduce the impact and improve partial functionality in some cases (American Occupation Therapy Association [AOTA], 2021).

There are many aspects in the lives of manual wheelchair users (MWU) that can have a distinct impact on their quality of life (QOL). Of particular interest in this review are the Community Mobility (CM) and Community Participation (CP) of the MWU as, according to a recent 2020 study by Rössler et al. (2020) the progression of Community Mobility and Participation (CMP) recovery post-injury directly impacts individuals’ wellbeing among their 59 participants. Additionally, Abou and Rice (2022b) study of 59 wheelchair users found that a higher rate of CM was associated with a lower risk of falling overall. The American Occupational Therapy Association (AOTA) defines CM as “moving around in the community and using public or private transportation, such as driving, walking, bicycling, or accessing and riding in buses, taxi cabs, or other transportation systems” (American Occupation Therapy Association [AOTA], 2021). Whereas CP was defined by Lachapelle and Austin (2014) as “the act of engaging in and contributing to the activities, processes, and outcomes.” Sweis and Biller (2017) review of the complications associated with SCI, a common cause of MWU, over the course of a year showed that these negative impacts “universally persist over time.” Furthermore, a 2018 study by Loyd et al. (2018) found that 53% of the 173 participants experienced a clinically significant decline in CMP after hospitalization. The participants in Loyd et al. (2018) study were over 65 years old, and all had been hospitalized for non-surgical medical reasons. This trend of decline in CMP after hospitalization continued to be shown as a 2023 study indicated that the CMP of wheelchair users over the age of 65 “reported more physical difficulties and were much less likely to go outside” over the course of the 8-year study of 7,026 participants (Nie et al., 2023).

Community Mobility and Participation directly impacts the wellbeing of MWU (Rössler et al., 2020), including reducing physical health, mental health (Selph et al., 2021) and the general QOL of MWU as further demonstrated by Hug et al. (2021) study of 500 patients (Hug et al., 2021). Not only are CMP vital to the wellbeing of MWU, but a 2018 study of 1,545 participants indicated that a majority of people think “that self-directed mobility is a fundamental right” (Logan et al., 2018). The importance of CMP became particularly apparent during the 2020 COVID-19 pandemic, which highlighted how much CMP could be impacted when individuals have externally imposed restrictions on movement as shown by Fortin-Bédard et al. (2022) in their 2022 analysis of 14 wheelchair users.

The primary goal of this review is to focus on relevant and recent articles related to assessing CMP of MWU, rehabilitation efforts, and factors that can impact the CMP. Studies focused solely on powered wheelchair use were excluded due to inherent differences between powered and MWU in physical activity levels, upper body strength, injury risk (da Silva Bertolaccini et al., 2022), and the ability to propel themselves. MWU are at an increased risk of developing upper extremity injuries due to repetitive strain and overuse of the shoulder joint (Ambrosio et al., 2005), as well as pressure sores and other wheelchair-related injuries (Warner et al., 2022). The MWU population is, therefore, essential to study to understand the unique challenges and limitations surrounding their CMP.

This review aims to examine how the rehabilitation of CMP for MWU is currently approached, what factors should be considered during rehabilitation, and where future research into the CMP of MWU could have the most potential to improve the QOL for MWU. Current methods of measuring CMP of MWU are examined, identifying their scope and limitations in a rehabilitation setting. Additionally, this review shall identify common barriers that can impact CMP and explore how they can be accounted for in future studies.

## 2 Materials and methods

The key search terms utilized to discover the reviewed articles were “Manual Wheelchair,” “Environmental Factors,” “Intrinsic Factors,” “Measurement Methods,” “Barriers,” “Long-Term,” “Lifestyle,” “Discharge,” “GPS Tracking,” “Community Opportunities,” “Rehabilitation,” “Continuous Rehabilitation,” “Community Mobility,” “BCI,” “Upper Limb Rehabilitation,” “FES,” and “Community Participation.” These terms were combined using Boolean operators “OR” and “AND” to enhance the search. For example, “Manual Wheelchair” was combined with “Community Mobility,” “Community Participation,” “Measurement Methods,” “Environmental Factors,” “Intrinsic Factors,” “Rehabilitation.” The terms directly related to the central question of this review were further enhanced through clarifiers like “Lifestyle,” “GPS Tracking,” and “Long-Term.”

The initial search was conducted through websites and search engines including PubMed, Google Scholar, ResearchGate, and Science Direct. From the discovered articles and online resources, a selection of 417 was obtained and considered for inclusion. After further examination and screening, 105 articles were included in the discussion.

**FIGURE 1:**
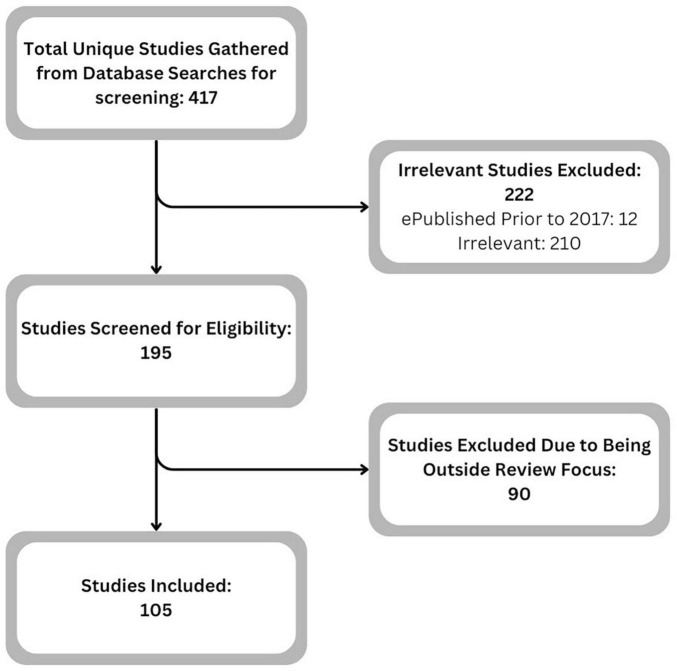
Selection of study flowchart.

The cutoff publishing date of 2017 for primary sources was selected as advances in research methodology change how research is conducted. Older research is likely to have already been integrated into clinical practice. Articles from before 2017 were included as additional support for the claims and assertions of the primary sources. The search was carried out until the end of September of 2023. The initial determination of the purpose and usefulness of each prospective article was done by screening the abstract and conclusion before examining the body. All articles included in this review were examined to ensure the information contained therein was timely, relevant, and could be effectively utilized within the scope of this review. Articles excluded were determined to be outside the focus of this review. The prospective articles included discussions on CMP, general mobility, rehabilitation, the use of BCI technology for upper limb rehabilitation, or factors that impacted CMP. Not every article selected was directly related to MWU, but every article selected for inclusion was chosen to support the review’s focus on MWU. A brief overview of the studies included in this review is presented in Supplementary Table 1, and a Selection of Study Flowchart can be found in Figure 1.

## 3 Methods of measurement for community mobility and participation

Thirty-three of the identified primary sources focused on or were related to the method of measuring the CMP of MWU. This assessment aimed to identify current best practices for measuring CMP, including assessment techniques already in clinical use alongside novel solutions from research. These methods can be divided into three general categories: Self-Surveys, In-Clinic Measurements, and Remote Measurements, as shown in Table 1.

**TABLE 1:** Methods of measurement.

Method of measurement	Measurement category	Measures
Life space assessment (Bayley et al., 2019)	Self-survey	CP
Wheelchair use confidence scale (Bayley et al., 2019)	Self-survey	CM
Functioning every day with a wheelchair (Sarsak, 2018)	Self-survey	CMP
Community integration questionnaire (Akyurek et al., 2019)	Self-survey	CP
Functional independence measure (Akyurek et al., 2019)	Self-survey	CMP
Wheelchair skills test questionnaire (Vader et al., 2019)	Self-survey	CMP
6-min push test (Damen et al., 2020)	Clinical measurement	CMP
VO2 max test (van der Westhuizen et al., 2017)	Clinical measurement	CM
Borg scale of exertion (van der Westhuizen et al., 2017)	Self-survey	CM
Reintegration to normal living index (van der Westhuizen et al., 2017)	Self-survey	CP
Wheelchair outcome measure for young people (Field and Miller, 2020)	Clinical measurement	CP
GPS tracking (Fillekes et al., 2019)	Remote measurement	CMP
Accelerometer (Bourassa et al., 2020)	Remote measurement	CM

CP, community participation; CM, community mobility; CMP, community mobility and participation.

### 3.1 Self-survey

Two self-survey methods of measurement for CMP come from Bayley et al. (2019) paper, the Life Space Assessment (LSA), which measures CP, and the Wheelchair Use Confidence Scale (WheelCon), which is used to measure CM (Bayley et al., 2019). The LSA asks questions on activity in five different aspects of life, ranging from inside the home to places outside of town, adding the results into a single sum score from each category. The Wheelcon asks for a confidence score from 0 to 100 on performing different activities and moving in different circumstances, with the total score being added into one combined number. However, the article does not directly assess subjects, instead basing findings on meetings and literature reviews with wheeled mobility experts and the World Health Organization’s (WHO) guidelines for wheelchair services (Bayley et al., 2019). The LSA surveys indicators like frequency of activities of daily living in the community, giving rehabilitation specialists an idea of the MWU’s involvement in their local community, whereas the WheelCon surveys indicators like wheelchair confidence under different circumstances (Bayley et al., 2019). WheelCon was also used by Giesbrecht and Miller (2017) in their 2017 study of 18 MWUs and their “confidence to operate the device safely and effectively.” In Giesbrecht et al. (2021) demonstrated their confidence in WheelCon when they designed their study protocol to measure the confidence of MWUs in their training program. Furthermore, a 2019 study by Sol et al. (2019) of 62 MWU indicated the WheelCon’s validity in measuring CM during their efforts to adapt it to the Dutch population. The WHO (Bascom and Christensen, 2017) supports the LSA and WheelCon as measurements of CMP, a testament to their validity and reliability.

Aside from the LSA and WheelCon, there are several other self-surveys which have been utilized to measure CMP. In 2018, Sarsak examined the satisfaction of 26 wheelchair users with their new wheelchairs by applying a novel method to measure CMP. Sarsak (2018) article utilized the Functioning Every Day with a Wheelchair (FEW) user survey, designed to measure participant’s functional independence, a strong indicator of CMP according to Anderson et al. (2003) study of 216 adults. A 2019 study by Akyurek et al. (2019) examined 270 wheelchair users in Türkiye in order to determine their CMP using the Community Integration Questionnaire to measure CP, the Functional Independence Measure to measure functional independence, and the Leisure Satisfaction Scale to measure satisfaction with leisure activities. Another 2019 trial by Vader et al. (2019) looked at 40 older MWU using multiple tests when examining CMP, including the WheelCon and Wheelchair Skills Test Questionnaire, another measure which assesses the physical capabilities of the MWU to determine their CMP.

Self-Surveys are the easiest to conduct, especially with large populations, as they require virtually no setup and have few limitations making them very popular. However, they lack precision and have common reliability issues that can impact how participants respond to self-survey questions. These issues can range from response bias to self-reporting biases, as participants may be inclined to exaggerate or not include everything. For example, a 2020 study by Yin and Tan (2020) found that compared with GPS data, self-reported CM levels in surveys are not accurate sources of the actual CM of the wheelchair user due to “limitations in recall ability and possibilities of perception bias.” This lack of precision and the issues faced make it necessary for any study utilizing self-surveys always to be mindful of how it can impact the results of their research. However, several methods, like the stochastic frontier estimation (Rosenman et al., 2011) and even following up with the participants via SMS text messaging (Brenner and DeLamater, 2016), have demonstrated the ability to mitigate the reliability issues associated with self-surveys at least partially. Furthermore, it is essential that, when using self-surveys, researchers ensure a comfortable environment and keep data confidential in order to receive the most accurate and honest responses.

### 3.2 Clinical measurement

One means of measuring CMP in the clinic is through physical activity tests, such as the 6-Min Push Test (6MPT). The 6MPT takes a previously utilized method for measuring non-wheelchair user CMP, gait speed which is typically measured through the 6-Min Walk Test (Beaverson et al., 2005; Lord and Rochester, 2005), and adapts it for MWU. Damen et al. (2020) 6MPT is based on the indicator of distance pushed in 6 min to measure MWU functional independence, a strong indicator of CMP (Anderson et al., 2003). Damen et al. (2020) used the 6MPT to evaluate 53 young individuals between the ages of 5 and 19 to verify its accuracy against standard in-clinic measurements for physical activity and concluded that “the 6MPT is a reliable, functional performance test on a vigorous level of exercise.” One thing to consider when utilizing a test like the 6MPT is that, according to a 2022 study by Andrews et al. (2022) MWU “who utilize their personal wheelchair demonstrate faster wheelchair propulsion speeds complimented by greater push frequencies” compared to unfamiliar wheelchairs.

One in-clinic means of measuring CP is physical fitness. This was examined in 2017 by van der Westhuizen et al. (2017) assessing the physical fitness of 60 MWU with SCI in an attempt to associate it with CMP. Physical fitness was measured via the VO_2_ max test, the 6MPT, and the Borg scale of exertion while CP was measured through the Reintegration to Normal Living Index (RNLI) (van der Westhuizen et al., 2017). The RNLI was recommended by the Academy of Neurologic Physical Therapy (McCombs et al., 2020) for use in measuring CP and has had its validity demonstrated in multiple previous studies (Harker et al., 2002; May and Warren, 2002; Hitzig et al., 2012; Mothabeng et al., 2012). Based on the data collected during their study, van der Westhuizen et al. (2017) concluded that there was “a relationship between physical fitness and community participation in [people with spinal cord injury] PWSCI.” To rehabilitation specialists, this finding indicates that measuring changes in the physical fitness of their patients would point to a corresponding change in CP, making assessment easier.

Another means of measuring the CP of pediatric wheelchair users comes from a 2020 study by Field and Miller (2020). In their study, Field and Miller (2020) evaluated the validity of a novel method for measuring CP, the Wheelchair Outcome Measure for Young People (WhOM-YP). By using a mixed methods study of nine therapists and nine wheelchair users ages 18 and under, they were able to show evidence supporting “WhOM-YP reliability and validity for measuring participation outcomes in daily life for young people” (Field and Miller, 2020).

Clinical measurements are precise, providing rehabilitation specialists with an accurate and reliable assessment of the CMP of MWUs. Furthermore, the gold standards for measuring physical activity and fitness, measurement metrics of CMP, are mostly in-clinic measurements. However, there are drawbacks to in-clinic measurement, namely that they only represent an instant in time and are not sensitive to daily changes and barriers that impact real-world CMP. This can result in conclusions that do not accurately reflect the day-to-day life of the population being examined. One potential way to mitigate this drawback is to closely simulate the conditions faced in day-to-day life when conducting in-clinic measurements.

### 3.3 Remote measurement

Remote measurement techniques using GPS or accelerometer data to measure CMP outside the clinic have been demonstrated to be feasible. Fillekes et al. (2019) study of 95 ambulatory participants found GPS tracking to be a valid means of measuring CM indicators. The indicators that Fillekes et al. (2019) identified and successfully tracked were extent of living space, quantity of out-of-home activities, time spent in active transport modes, stability of life space, elongation of living space, and timing of mobility. Other studies such as a 2017 study by York Cornwell and Cagney (2017) have also successfully utilized GPS data to examine CP. Through examining GPS locational data of 60 older adults, Cornwell and Cagney were able to evaluate how much of their participants’ time was spent outdoors doing different activities in the local community (York Cornwell and Cagney, 2017). Further support for using GPS to measure CMP can be found in a 2020 study by Zhu et al. (2020) which successfully tracked the trip frequency and duration, indications of CMP for their 54 older adults.

During the COVID-19 pandemic two studies were conducted by Sun et al. (2021) and Nanda et al. (2022) on the use of phone GPS to track CMP. Nanda et al. (2022) study examined a large set of mobile phone data and tracked their CMP through locations visited in relation to the spread of COVID-19. In a similar fashion Sun et al. (2021) also obtained and examined a large subset of phone GPS data to track the CMP of the phone users through their time away from home to relate it to how COVID-19 was progressing in the community. This work by both studies demonstrates the validity and applicability of using GPS for the remote measurement of CMP. While less common, accelerometers have also been used in the remote assessment of CMP. In 2020, using an accelerometer in the form of actigraphy, a non-invasive means of measuring cycles of activity and rest, Bourassa et al. (2020) examined and compared remotely measured physical fitness for 28 MWUs against their clinically measured heart rate and perceived exertion. Bourassa et al. (2020) concluded that using the actigraphy alongside perceived exertion “could be an easy and reliable method to measure the intensity of real-world activities.”

When it comes to applying remote measurement, there are drawbacks to consider. For instance, a 2018 study by Boissy et al. (2018) found that, in their sample size of 75 participants, “Wearability and usability of the devices used to record the data affect compliance and data quality,” (p11) the solution to which would necessitate compromising the continuous sampling of GPS and accelerometer data. Furthermore, a 2019 study by Seymour et al. (2019) surveyed 21 community-based rehabilitation workers in Uganda, finding that one struggle faced by wheelchair users was the lack of quality wheelchairs. Additionally, Fillekes et al. (2019) study addressed privacy concerns with GPS units, suggesting that researchers should be transparent with the participants about data that is being collected as well as the potential risks involved regarding their personal information. Finally, accelerometers such as those used in inertial measurement units can have significant deviations in accuracy from gold standards, according to a 2022 study by Henschke et al. (2022).

Remote Measurement is a crucial tool for rehabilitation therapists when working with MWU. It allows for a large amount of data to be collected almost anywhere in the world and analyzed with little trouble for the patient. There are downsides to be considered, from privacy concerns as to how the gathered data is being used to a lack of precision of the data and an inability to record the subjective experience of the participant. Remotely measuring CMP has the potential to give more accurate results in the actual community compared to in-clinic measurements and surveys. As Jang et al. (2023) found in their 2023 study of 20 participants, “community-based testing may provide a better reflection of everyday performance.” By clearly and concisely communicating why data is being gathered and what it is being used for and ensuring that their data will only be used within the scope of the study, researchers can protect the privacy of the participants’ remote measurements.

## 4 Factors impacting community mobility and participation

**FIGURE 2:**
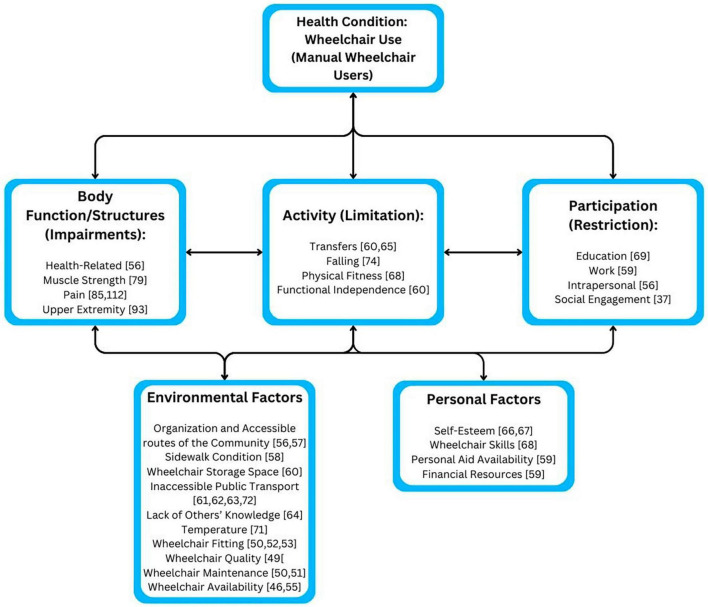
International classification of function, disability and health model of factors affecting CMP for MWUs.

Community Mobility and Participation are affected by community, personal, and environmental factors in the lives of MWU. Twenty-four primary sources were identified and examined for this aspect of CMP. An International Classification of Function, Disability, and Health model that gives an overview of factors affecting CMP for MWUs can be found in Figure 2.

### 4.1 Community factors

One significant community factor affecting CMP of MWU is the quality of the mobility device. This was examined in 2018 by Magasi et al. (2018) who surveyed 250 individuals who utilized wheelchairs. Their conclusion was that “Mobility device quality plays an important role in participation outcomes” (Magasi et al., 2018) with 20% of all variance in participation reported to come from the device quality, specifically repairability, ease of maintenance and reliability (Magasi et al., 2018). Oldfrey et al. (2023) study supports Magasi et al. (2018) conclusions on repairability as their review of studies in Kenya, Uganda, Sierra Leone and Indonesia concluded that one of the often overlooked aspects of CMP for MWU is the repair of the wheelchairs. Ferretti et al. (2022) review of 18 studies in 2022 also highlighted the importance of wheelchair maintenance for CMP.

Others have also investigated the impact of wheelchair quality on the CMP of MWU. One 2017 study by Bazant et al. (2017) surveyed 852 participants in Kenya and the Philippines to see how wheelchair service provision affected wheelchair-use-related outcomes. Their survey found that when the wheelchair was fit to the user, they had higher odds of greater CM (Bazant et al., 2017). A more recent 2022 study by Nuri et al. (2022) reached the same conclusion when they surveyed 376 wheelchair users in Bangladesh and found that, alongside poor-quality roads, the fitting of their wheelchair was affecting their CMP. Even more support comes from Mattie et al. (2019) study in 2019 which found that, when it came to CMP, “adjustable seating can have a significant impact on ultralight wheelchair users.”

Not only is the quality of the wheelchair important, but the availability of wheelchairs can also have a large impact on CP. Seymour et al. (2019) investigated what challenges 21 community-based rehabilitation workers faced in Uganda, concluding that one of the biggest challenges was in providing wheelchairs. A 2022 study by Gowran et al. (2020) also addressed wheelchair provision, surveying 281 wheelchair users in Ireland. The results of the survey showed that, while anxiety and a sense of insecurity were identified as factors impacting CMP, wheelchair and seating assistive technology provision was by far the most important and was considered to be a basic human right.

Other barriers to accessibility in the community also impact an individual’s CMP. A 2020 study by Hansen et al. (2020) examined 181 Danish participants with mobility impairments to see what barriers were prevalent and had the most impact on CMP. Their study found that, while intrapersonal and health-related barriers were the most prevalent, organizational and community barriers, such as accessibility to buildings and locations, were the most severe (Hansen et al., 2020). Another example comes from a 2020 study by Prescott et al. (2020) that surveyed 78 individuals, finding that the most important modifiable factors affecting CMP are related to the accessibility of the community. Ultimately, they concluded that “a dense neighborhood with accessible routes to accessible buildings with inclusive programs and services should be the goal” (Prescott et al., 2020). The type of neighborhood called for would provide shortened routes for MWU and programs incentivizing participation. This is further supported in a 2021 study by Gharebaghi et al. (2021) who used trip data to show how poor sidewalk conditions could significantly impact the CMP of individuals with motor disabilities. This highlights the importance of ensuring that new developments and existing neighborhoods are up to code regarding accessibility. Furthermore, it demonstrates the importance of current accessibility laws and standards doing their utmost to ensure that MWU and others facing similar challenges can participate and maneuver through their communities. The lack of wheelchair-friendly infrastructure in workplaces and prevailing societal biases can limit the options that MWUs have when it comes to finding a stable job. This not only limits employment opportunities but can also contribute to income disparities within the wheelchair-user community (Toro-Hernandez et al., 2020).

Others such as Koontz et al. (2020) have also studied the effect of community barriers on CMP. Koontz et al. (2020) surveyed 112 wheelchair users, asking them what barriers hindered them during independent transfers. According to the results of that survey, the lack of spaces for easy transfer, grab bars, transfer aids and storage space for their wheelchairs could significantly limit the participants participation in their community (Koontz et al., 2020). Barriers in the form of transportation were of particular focus to Bezyak et al. (2017) study, which surveyed 4,161 individuals with disabilities and found that the most common barriers to CP in public transportation included inaccessible stops, stations, and the driver’s attitude or lack of knowledge. Reinforcing this is a 2017 study by Grills et al. (2017) which, surveying 2,431 adults, identified that some of the most significant barriers to individuals with disabilities were “lack of information, transport, and physical inaccessibility.” This can make it difficult for MWU to plan their accessible routes in their daily commutes. Two years later in D’Souza et al. (2019) published an article highlighting their examination of what their 48 wheelchair user participants thought were the major barriers to CM when using a bus for public transit. The largest issues identified by the wheelchair users were the difficulty in maneuvering inside the bus as well as in getting on and off (D’Souza et al., 2019). Public transportation is relied upon by large numbers of people worldwide for their day-to-day lives. It is critical to promote and not hinder CMP for MWU, which is crucial for their QOL. Inaccessible public transportation options often force individuals to resort to costly alternatives, such as specialized taxis or rideshare services. The limited availability of accessible transportation can also hinder travel for job opportunities (Toro-Hernandez et al., 2020).

The lack of knowledge in the community when it comes to wheelchair transfers was further highlighted by Holt et al. (2021) in their 2021 study. Specifically, Holt et al. (2021) identified that one of the biggest issues that female wheelchair users faced for CMP in gynecologic care was the lack of knowledge that the healthcare providers had, making the transfer to the exam table more difficult. An earlier study by Barbareschi and Holloway (2019) also highlighted the cruciality of wheelchair transfers for the CP of MWU from the interviews conducted with 11 wheelchair users and 4 occupational therapists.

These community barriers collectively form a vast, complex web of challenges faced by not only MWUs but also those seeking to improve accessibility within a community. These factors and their interactions must be considered and thoroughly investigated to ensure that the multifaceted nature of CMP for MWU is addressed. This could potentially be addressed by educating people about the needs of MWUs in order to have a high QOL through peer-based community programs and providing support for policies to improve that QOL.

### 4.2 Personal factors

Factors which are more personal and intrinsic can also significantly impact the CMP of MWU. This was highlighted in De Serres-Lafontaine et al. (2023) study on the CP of MWU in Tanzania. Their examination of 10 MWU indicated that personal self-efficacy and self-esteem significantly impacted CP and “it is ‘one of the many challenges people with disabilities face” (De Serres-Lafontaine et al., 2023). The importance of self-efficacy and self-esteem was further highlighted by Abou and Rice in their 2022 study of 59 wheelchair users. Abou and Rice found that both wheelchair skills and depression could significantly influence the CP of the wheelchair users (Abou and Rice, 2022a). Wheelchair skills were also found to be “associated with participation” by Ferretti et al. (2022) in their 2022 systematic review of 18 studies. Another 2022 study by Silveira et al. (2022) directly examined 26 SCI MWU with the Wheelchair Skills Test Questionnaire, seeking to see what role the wheelchair skills of a MWU played in their CP. Silveira et al. (2022) concluded that not only were wheelchair skills directly related to CP, but they were also “significantly associated with fitness in persons with SCI.”

Another study of 32 participants in Toro-Hernandez et al. (2020) highlighted other personal factors that could impact CMP, specifically financial resources, inaccessible housing, and lack of personal aid. The cost associated with acquiring assistive technologies or modifying workspaces or places may not be fully covered by existing policies. Recognizing and addressing these financial impediments is essential for fostering inclusivity and enhancing the quality of life for MWU. Future interventions and policies should be crafted with a keen understanding of these challenges to ensure equitable opportunities for all individuals, regardless of their mobility status. In Hansen et al. (2021) examined the perception of barriers of 181 MWU, finding that education could significantly impact the CM for MWU, requiring “special consideration and resources to overcome distinct physical activity barriers.” There is one additional personal factor of CMP that should be considered, specifically the risk of falling. Not only does falling have the potential to further injure the MWU, but even the fear of falling can negatively impact the MWU’s CP according to Sung et al. (2020) examination of 54 wheelchair users in 2020.

### 4.3 Environmental factors

The environment where a MWU lives can also have a significant impact on CM. This was highlighted by a 2018 Canadian study by Borisoff et al. (2018) which examined 11 individuals and concluded that snow and freezing temperatures could significantly decrease the CM of MWU compared to summertime. Another 2017 study by Ripat et al. (2017) examined the impacts of different weather patterns on three Canadian wheelchair users over the course of a year through interviewing them once a month. They found that winter weather conditions created significant CP challenges for their participants, but that it had more of an effect on what choices were available to the wheelchair user, rather than the overall level of CP (Ripat et al., 2017). Instead, the most consistent factor for CP was the availability of vehicular transportation (Ripat et al., 2017).

Understanding the factors that influence the CMP of MWUs is critical. From the community, to personal, to the very environment, every aspect of a MWU’s life can impact their CMP. Not only do these factors provide insights into the underlying issues facing MWUs, but they also give therapists the knowledge of where rehabilitation should be focused to have the most direct impact.

## 5 How rehabilitation can improve community mobility and participation

Rehabilitation is a widespread and commonly used method for improving the lives of MWU. There are many forms and practices that are taken when prescribing rehabilitation therapy to MWU. Forty-eight of the identified primary sources looked at different types of rehabilitation for improving the CMP of MWU.

### 5.1 Stability and upper limb functionality

An article by Rice et al. (2019) studied 20 MWU who had experienced falls and assessed how rehabilitation could help them recover. Their results found that those who most needed assistance through rehabilitation also had the lowest CP (Rice et al., 2019). The fear or risk of falling has been shown to be a significant factor affecting CP (Sung et al., 2020), and one of the greatest factors that is associated with the chance of falling is stability (Singh et al., 2020). In 2018, electrical stimulation was shown to be effective at improving MWU stability by Armstrong et al. (2018) study of three participants. Functional electrical stimulation (FES) has also been shown to be effective generally in the rehabilitation of muscle function, including the upper extremities. This was demonstrated by Chamran et al. (2023) in their 2023 examination of 12 MWU through tracking the trajectories of the upper limb and muscle activations, which showed advantages in generating optimal joint torque and accurate trajectory tracking.

A 2017 review by Rice and Rice (2017) examined articles on physical rehabilitation and concluded that the “Preservation of [upper limb] UL function and pain prevention among full-time MWU is critical to promote high levels of quality of life and community participation.” Another aspect of rehabilitation to consider is the effort to improve muscle flexibility, vital to upper-limb function. This was highlighted in a 2017 study by Finley and Ebaugh (2017), which assessed muscle flexibility through measuring pectoralis minor muscle length and extensibility in 22 MWU compared to the duration of wheelchair use, finding an inverse correlation between duration and flexibility.

The use of brain-computer interfaces (BCI) when rehabilitating or training the upper arms, a crucial aspect for MWUs CMP, has great potential. A 2018 study by Remsik et al. (2018) successfully demonstrated with their 14 participants that the use of a BCI intervention helped improve their mobility, and strength in their arms. Not only that, but Bockbrader (2019) review further highlighted the effectiveness of BCI when it comes to the restoration of upper limb sensorimotor and hand function. Another review of the effect BCI can have on upper limb rehabilitation was published in 2018 by Cervera et al. (2018) which examined the contents of 26 articles studying a total of 235 stroke patients and the use of BCI in their rehabilitation routine. Cervera et al. (2018) findings indicated that “BCI technology could be an effective intervention for post-stroke upper limb rehabilitation.” Nojima et al. (2021) also reviewed literature on BCI used for aiding rehabilitation in 2021. During their review of 16 articles involving 382 stroke patients, Nojima et al. (2021) reached the conclusion that “this meta-analysis suggested that BCI-based training was superior to conventional interventions for motor recovery of the upper limbs in patients with stroke.” However, they did acknowledge that their results were not conclusive due to a high risk of bias and heterogeneity (Nojima et al., 2021). Another study in 2018 also studied the usefulness of BCI for rehabilitation and training of upper limbs in stroke patients. Nishimoto et al. (2018) conducted a trial of a BCI therapy program for 26 stroke patients. Their findings indicated that their performance scores for upper limb motor activity “improved significantly after brain-machine interface training” (Nishimoto et al., 2018). Yet another example comes from a 2022 study of 12 participants by Shen et al. (2022) which demonstrated the effectiveness of BCI when paired with other rehabilitation methods in improving upper limb mobility.

Brain-computer interfaces technology is also accessible enough that it can be used at home, with one 2019 study of 20 participants concluding that “Portable [Neurofeedback] NFB is a feasible solution for home-based self-managed treatment of [central neuropathic pain] CNP” (Al-Taleb et al., 2019). Al-Taleb et al. (2019) also discovered that the BCI NFB had few side effects and helped users to have control over their pain. Furthermore, BCI intervention was demonstrated to “promote long-lasting retention of the early induced improvement in hand motor outcome” (Mattia et al., 2020). By Mattia et al. (2020) in their 2020 trial with 48 stroke patients. Not only can BCI technology improve rehabilitation outcomes, be accessible at home and promote long-lasting impact, but it has the potential to directly improve the QOL of MWU. According to a 2020 mini-review by Belkacem et al. (2020) there is a plethora of potential applications for BCI technology which could improve QOL for elderly individuals and those needing aid such as MWU.

### 5.2 Neuroergonomics approaches

Utilizing BCI alongside electroencephalography (EEG) was shown by Carino-Escobar et al. (2019) to have the potential to aid rehabilitation specialists during their sessions with wheelchair users. Their 2019 study was able to successfully demonstrate how, with 9 stroke patients, EEG can be used to improve prognosis accuracy and BCI cortical activity targets (Carino-Escobar et al., 2019). During a systematic review in 2021 of 18 studies on BCI, virtual reality (VR), and EEG for rehabilitation, Camargo-Vargas et al. (2021) concluded “that using EEG signals, and user feedback offer benefits including cost, effectiveness, better training, user motivation.” Using an EEG in conjunction with a BCI during rehabilitation can be accomplished while keeping the EEG use non-invasive (Zhuang et al., 2020).

The combination of BCI alongside other forms of rehabilitation, including FES, has been shown to have the potential to yield greater results than the individual parts in aiding the functionality of upper-limbs according to Cajigas and Vedantam (2021) review of literature at the time. This was also the focus of a 2021 study by Samejima et al. (2021) who specifically looked at the potential for BCI paired FES treatments to restore upper limb functionality by first studying its affect in rats with SCI, finding that it was effective. In humans, another 2021 study, this one by Jovanovic et al. (2021) similarly examined how a BCI paired FES treatment would help 5 SCI patients. Their findings concurred with those of Samejima et al. (2021) concluding that their “new [brain–computer interface-triggered functional electrical stimulation therapy] BCI-FEST intervention is safe, feasible, and promising for the rehabilitation of reaching and grasping after SCI” (Jovanovic et al., 2021). A 2020 case study by Jovanovic et al. (2020) examined the effect of using BCI to trigger FES therapy for a 57-year-old male stroke survivor. They used their case study to demonstrate the effectiveness, safety and viability of using BCI with FES therapy (Jovanovic et al., 2020). In Jervis-Rademeyer et al. (2022) also examined the potential for BCI triggered FES therapy to aid in the rehabilitation of upper limbs in SCI patients. Jervis-Rademeyer et al. (2022) interviewed a total of 6 therapists to ascertain perspectives on BCI triggered FES therapy and identify facilitating and limiting factors affecting delivery of the therapy. The factors that they identified for intervention were education, training, a support network, or mentors, and restructuring the physical environment (Jervis-Rademeyer et al., 2022).

Other integrations of BCI have also been shown to be effective, such as a 2017 study by Spicer et al. (2017) who tested 12 older adults to demonstrate the safety and effectiveness of using BCI in conjunction with VR to promote upper limb motor recovery. VR has further been proven useful alongside BCI in improving rehabilitation by Lakshminarayanan et al. (2023b). In their 2023 study of 15 participants with and without VR integration, they found that using the VR system “enhances brain rhythmic patterns and provides better task differentiation” (Lakshminarayanan et al., 2023b). Another 18 healthy participants were examined in 2018 by Achanccaray et al. (2018) whether the integration of VR feedback for a BCI interface could aid in upper limb rehabilitation. Their study found that when using the BCI system in partner with VR, the participants experienced “a sustained and more intense premotor cortex activation,” indicating that it had the potential to improve rehabilitation efforts (Achanccaray et al., 2018). Similar technology comes from Lakshminarayanan et al. (2023a) when they examined the use of Tactile Imagery (TI) as opposed to Motor Imagery (MI) alongside BCI. MI is a common means of rehabilitation for improving muscle strength after a stroke. They investigated the effect of TI on BCI for 12 healthy individuals during a rehabilitation exercise, concluding that “compound tactile imagery can be a viable alternative to MI for BCI classification” (Lakshminarayanan et al., 2023a).

Using BCI with both VR and FES is yet another combination of techniques which can be used to help the rehabilitation process for the upper limbs. For example, in Sebastián-Romagosa et al. (2020) study of 51 stoke patients, they found the combination of BCI, VR and FES in a rehab treatment “was effective in promoting long lasting functional improvements in the upper extremity” (Sebastián-Romagosa et al., 2020). In Vourvopoulos et al. (2019) closely examined a clinical case of a 60-year-old stroke patient to see how the integration of VR, EEG, and BCI intervention impacted their CMP. The use of the integrated intervention resulted in measurable improvements to upper extremity mobility scores as well as in brain activation.

### 5.3 Physical fitness

Physical fitness has also been shown to be essential for the CMP of MWU. An examination of rehabilitation impacts on CMP came from Katri Maria et al. (2021) study on home-based rehabilitation for older individuals following hospital discharge. The study’s examination of the 117 participants found that, while rehabilitation did not improve the participants’ physical activity levels (Katri Maria et al., 2021), it did find that the best time to evaluate mobility-restricted individuals was during hospitalization, as “Pre-admission mobility may determine the response to the largely counseling-based rehabilitation program” (Katri Maria et al., 2021). Physical fitness has also been shown to improve scores in wheelchair skills and confidence (Kirby et al., 2018), both key factors for CMP of MWU in their own right and demonstrating the importance of rehabilitation improvement and preservation of physical fitness for MWU. This was further supported by Sol et al. (2021) in their 2021 study of 60 MWU, concluding from their examination of different training and rehab methods that “A combination of exercise and [Wheelchair Mobility Skills] WMS training appears to have significant positive long-term effects on [physical activity] PA, WMS, confidence in wheelchair mobility, and (an)aerobic performance in youth using a manual wheelchair.” Another example of using rehabilitation to promote physical fitness in MWU was given by Canori et al. (2023) survey of 26 participants which found that improving a patient’s CP could “potentially improve motivation for PA.” The preservation of physical fitness was shown to be achievable through active rehabilitation in Lipert et al. (2021) study of how rehabilitation affected physical performance for their 42 MWU participants.

### 5.4 Training programs

In 2021, a critical review by Livingstone and Paleg (2021) examined over 200 studies for children with mobility disorders and found that intervention and rehabilitation, like strength training, stretching programs, and wheelchair use training, could improve the MWU’s participation. In Hossain et al. (2023) conducted a cross-sectional survey of 90 MWU to see the effect of wheelchair skills training. They found that “wheelchair skills training enhance[s] confidence and participation among people with spinal cord injury” (Hossain et al., 2023). A 2020 systematic review of 4 studies by Willig et al. (2020) also showed promise in using training programs. Their findings indicated that “resistance training improved functional independence while both types of exercise (aerobic arm-ergometer and resistance training) included positive effects on quality of life” (Willig et al., 2020). One more example of the success of training programs can be found in a 2020 article by Pellichero et al. (2020) which studied 11 individuals and conducted rehabilitation training programs to improve satisfaction and self-sufficiency. Their study found that all the participants “expressed satisfaction and quality of participation with the WheelSeeU program that increased autonomy, improved (Manual Wheelchair) MWC mobility and self-efficacy, and enhanced social connectedness” (Pellichero et al., 2020). Charlton et al. published a pilot study in 2021 which sought to explore how a wheelchair skills training program could affect performance, confidence and CP for MWU. Using a sample size of 11 participants, Charlton et al. (2021) were able to show that “The Wheelchair Skills Training Program can improve wheelchair performance, confidence and frequency to support enhanced safety, independence and quality of life for people with lower limb amputations.” This sort of training program can be accomplished both in clinic, and at home, as shown by Gauthier et al. (2018). Their study of 11 MWU sought to examine the impact of both high-intensity interval training (HIIT) and moderate-intensity continuous training (MICT) programs at the MWUs’ homes. Ultimately, Gauthier et al. concluded that both the HIIT and MICT programs could be safely used at home with no risk of injury, though some pain could be experienced (Gauthier et al., 2018).

### 5.5 Community/peer-based programs

Community peer-based rehabilitation program have been found to be effective for improving the CMP of wheelchair users. In Divanoglou et al. (2019) studied 17 individuals with SCI using a training program called Active Rehabilitation (AR), which provided opportunities for peer-led, community-based rehabilitation. Their results found that “the peer-based program AR can play an important role in promoting physical independence, wheelchair mobility, and injury-management self-efficacy in community-dwelling individuals with SCI” (Divanoglou et al., 2019). A further example for the effectiveness and importance of community-based rehabilitation comes from a 2019 study by Madsen et al. (2020) They reviewed nine articles on outdoor and community-based rehabilitation programs and reached the conclusion that the programs “seemed to empower participation opportunities and social inclusion of people with disabilities” (Madsen et al., 2020). Additional support comes from a 2022 pilot study that assessed 8 wheelchair participants and found that “a community-based peer-led approach to wheelchair skills training seems promising for improving wheelchair outcomes” (Ouellet et al., 2022).

One 2017 article by Best et al. (2017) sought to examine a novel peer-led MWU training program called the Wheelchair training Self-Efficacy Enhanced for Use (WheelSeeU), which consists of tasks to challenge MWU self-efficacy, examined 40 MWU to test the WheelSeeU program and its effect on their self-efficacy. The findings of Best et al. (2017) showed that “WheelSeeU is an innovative and feasible approach for providing MWC (Manual WheelChair) training to older adults that is accessible beyond initial rehabilitation without increased clinician burden.” Another study by Miller et al. (2019) also examined how the peer based WheelSeeU training program could improve the CP and skills for MWU. During their trial, Miller et al. examined the effect on 40 older adults, finding that “Compared to an active control group, WheelSeeU did not have a greater effect on wheelchair skills capacity.” However, they also stated that it should not be dismissed as it had the potential to be refined such that it could “potentially improve wheelchair skills performance and satisfaction with participation in meaningful activities” (Miller et al., 2019). In 2019 Giesbrecht and Miller (2019) examined 18 MWUs to see how a wheelchair skills training program using mobile application technology called mHealth would affect them. During their study they found that the “participants demonstrated good program adherence and clinical benefits were evident in community participation and wheelchair self-efficacy” (Giesbrecht and Miller, 2019).

### 5.6 Propulsion technique

Other forms of rehabilitation focused on improving MWU propulsion techniques and efficiency can also improve CMP. One study by Leving et al. (2019) found that through rehabilitation of their 8 MWU over 6 weeks, as measured by submaximal exercise tests, the mechanical efficiency of the more experienced MWU was higher than the less experienced MWU, indicating an increase in fitness (Leving et al., 2019). The improvement of wheelchair performance can also be aided using VR. Yang et al. (2021) examined the feasibility and efficacy of a VR simulator for training propulsion performance in 20 MWU. Their study found it an attractive, novel experience and found no significant difference in wheelchair propulsion kinematics between the VR simulator and clinical measurements (Yang et al., 2021). They concluded that using the VR simulator could enhance wheelchair maneuverability experiences (Yang et al., 2021).

## 6 Future work

Significant progress has been made in recent literature on techniques to assist MWU in improving their CMP and general QOL. However, many opportunities for future research exist to further support QOL of MWU. One example of a deficiency in current research that should be addressed is how most of the effort in upper-limb rehabilitation research and development has been aimed at non-SCI injuries, highlighted by Grampurohit et al. (2021) in their 2021 scoping review of upper extremity research focus areas.

Repairability and the fitting or adjustability of wheelchairs has proven to be a major factor that can impact the CMP for MWU (Mattie et al., 2019; Nuri et al., 2022; Oldfrey et al., 2023). To ensure the highest QOL for MWU, the repairability and maintenance of wheelchair designs as well as ensuring a fitted wheelchair or ease of adjustment for the wheelchair ought to be a major concern in all future design work (Mattie et al., 2019; Nuri et al., 2022; Oldfrey et al., 2023). The ease of adjustment for wheelchairs is especially important for pediatric wheelchair users as they are likely to need a much wider array of adjustability to effectively utilize their mobility assistive device as they rapidly grow from toddlers to young adults.

While our current study has provided multiple important factors affecting CMP for MWU, a gap exists in understanding how these challenges may vary across different age groups. Childhood and adolescence developmental stages introduce distinctive challenges that may impact mobility differently compared to adulthood. Factors such as growth and development, changing social dynamics, and evolving caregiving structures may contribute to a diverse set of obstacles for pediatric wheelchair users. As they grow, age-appropriate wheelchair modifications are critical for their CMP (Field and Miller, 2020). In contrast, adults navigating mobility challenges may encounter issues related to employment, independent living, and community engagement (Toro-Hernandez et al., 2020) distinct from those faced during childhood and adolescence. By addressing this age-specific gap in the comparison literature, future research has the potential to inform targeted interventions and policies that cater to the unique needs of adult and pediatric wheelchair users. This nuanced understanding is crucial for developing holistic strategies encompassing individuals’ entire lifespan relying on manual wheelchairs.

Considering recent positive outcomes from BCI, FES, and VR research, there is an opportunity to develop applications of the technology to support enhanced CMP for MWU. Current efforts have mostly been in upper limb rehabilitation, which, while important for the CMP of MWU, are only one aspect that must be considered by researchers.

If tailored for MWU, new technologies such as Geospatial Assistive Technologies (GATs), which combines route planners with a navigation system to plan and execute movements, have the potential to significantly aid MWU in their day-to-day CMP. This was made particularly apparent by Prémont et al. (2019) in their 2019 study of 17 MWUs which provided “useful data to improve GATs and broaden the concept of compatibility among users and specific-use situations to ensure usability.” A further area of deficiency which should be addressed was highlighted by Canori et al. (2023) in their 2023 survey of 26 SCI patients. Specifically, Canori et al. realized that physical activity motivational platforms, which they found to be beneficial for the CMP of MWU, “are not tailored toward wheelchair-users.” Considering the potential improvements to CMP and QOL such platforms have, ensuring that they are better geared for MWU should be examined further.

Wheelchair transfers have been shown to be a significant factor impacting the CMP of MWU, and they can be difficult for a MWU. In Barbareschi and Holloway (2019) interviewed 11 wheelchair users, concluding that while transfers “were described as gateways to independence that grant opportunities and community participation,” the skills required “are difficult to acquire and the concept of correct technique, although really important, is often poorly defined” (Barbareschi and Holloway, 2019). Barbareschi and Holloway (2019) also concluded that future research and collaborative effort would be needed to improve the transfers for wheelchair users. Additionally, knowledge for transfers of MWUs needs to be effectively conveyed both to the MWUs themselves as well as healthcare professionals and others who could aid in the transfer.

Another common deficiency that is plain to be seen across most of the research into the CMP for MWU is the lack of longitudinal studies. Most researchers that examine means of improving CMP for MWU only follow up after a short period of time (Achanccaray et al., 2018; Remsik et al., 2018; Carino-Escobar et al., 2019; Vourvopoulos et al., 2019; Sebastián-Romagosa et al., 2020). This is a significant gap in the research as any gains made through rehabilitation techniques are meant to be long-term, but there is a staggeringly low amount of research into what extent this occurs. A study which utilizes a remote measurement method such as GPS with continuous tracking of CMP of MWU long term would reveal important information across different seasons, environmental changes, travel patterns, and behavioral trends. More research and studies need to be conducted to ensure that any improvements from rehabilitation techniques, especially novel ones, are maintained long-term.

Finally, there is potential for mobile smartphone applications (apps) to become useful for rehabilitation therapists as well as MWUs. By using the GPS systems built into most modern phones, an app could provide an easy means of tracking CMP for MWU. Phone apps have already been used by MacGillivray et al. (2019) in their 2019 feasibility study to allow SCI patients to self-manage their rehabilitation and discharge. Another study by Marco-Ahulló et al. (2021) also used phone apps to track the energy expenditure of their 12 SCI patients. Clearly, apps have potential in rehabilitation applications. However, there are issues with current phone app designs that need to be addressed, namely they are not well optimized for use by MWU as an assistive technology. In 2020, Armstrong-Wood et al. (2023) interviewed 39 SCI patients, asking them what challenges they face when using their smartphone. They found that there were large deficiencies in information and support for phone assistive technology, alongside privacy concerns and a regard for current phone assistive technology being “overpriced, poorly design[ed] and lacking the voices of people with disabilities” (Armstrong-Wood et al., 2023). Further research and implementation for smartphone-based assistive technology should address these challenges and concerns.

## 7 Limitations

One limitation of this review stems from the nature of the topic. As CMP of MWU is a relatively understudied topic, there is not as wide an array of available sources to draw upon as desired. Further, assessment technologies and techniques are frequently changing, necessitating a shorter window of time for the review. Therefore, the primary focus of this review was on articles published during and after 2017 to maintain a modern, current perspective on methods, factors, and rehabilitation of the CMP of MWU.

## 8 Conclusion

By utilizing best measurement practices to assess CMP of MWU and considering key factors affecting the MWU’s life, rehabilitation practitioners are better equipped to meet the needs of those under their care. Surveys like the LSA, and the WheelCon or physical fitness, GPS, and accelerometer data tracking enable rehabilitation specialists to assess the CMP of MWU accurately. Additionally, by considering the climate, accessibility barriers in the local community, and personal factors, not only can realistic goals be set for rehabilitation, but training and resources can be justified to support the MWU. Ultimately, through early assessment and rehabilitation within the clinic and in the local community, CMP can be improved for the MWU. Future examination of the efficacy of GPS and accelerometer data as a method of tracking CMP will provide useful insight and crucial information for rehabilitation practitioners to improve the rehabilitation and QOL of those under their care. Additionally, expansion of knowledge in the neuroergonomic approach to the CMP of MWU will enable therapists to have a more effective array of tools at their disposal and support personalized rehabilitation planning for patients.

## Author contributions

GF: Writing – original draft, Writing – review and editing. MG: Writing – review and editing, MR: Writing – review and editing. JR: Writing – review and editing.
