# Supplementary material for: Community mobility and participation assessment of manual wheelchair users: a review of current techniques and challenges
Source: Front Hum Neurosci. 2024 Jan 5;17:1331395. doi: 10.3389/fnhum.2023.1331395 (PMC10796510; doi:10.3389/fnhum.2023.1331395)
Supplement: Supplementary file 1 [file Table_1.docx]

**Table 1:** Overview of Key Source Articles

| **Author** | **Synopsis** | **Type of Study** | **Cohorts of Interest** | **Data Collection Method** | **Outcome** | **Drawbacks** |
| --- | --- | --- | --- | --- | --- | --- |
| **Methods of Measurement for Community Mobility and Participation** | | | | | | |
| Bayley et al. 2019^16^ | Examination of indicators relating to rehabilitation | Literature Review | N/A | LSA, WheelCon | LSA and WheelCon shown to be effective means of measurement | Lacks participant data |
| Giesbrecht & Miller  2017^17^ | Examining feasibility of wheelchair skill training for older adults | Cohort Study | Manual wheelchair users | Survey | Lends support to Physical Activity improving community mobility and participation | Missing data |
| Giesbrecht 2021^18^ | Outlining Study Protocol for wheelchair training program | Study Protocol | Manual wheelchair users | Skill Assessment,  Survey | Lends support to Physical Activity improving community mobility and participation | No data, merely a protocol |
| Sol et al. 2019^19^ | Examines the development of the WheelCon-Mobility Dutch Youth a questionnaire that access the confidence of in wheelchair mobility of Dutch Youth | Intervention Study | Wheelchair users | WheelCon-Mobility | Confidence in wheelchair mobility | N/A |
| Bascom GW, Christensen KM; 2017^20^ | Examining the impacts that limited transportation access can have on Community Participation on people with disabilities | Cohort Study | Individuals with disabilities | Survey / Questionnaire | Provided basis for considering access to transportation as a factor that can impact community participation | N/A |
| Sarsak, 2018^21^ | The measurement of satisfaction and functional independence of wheelchair users | Descriptive quantitative research study | Wheelchair users | Survey (FEW) | Showed that surveys such as the FEW self report survey had the potential to yield data that could be used to help improve wheelchair user rehabilitation | Homogeneous sample |
| Akyurek et al., 2019^23^ | Examines the factors that are associated with community participation of people with disabilities | Survey Study | Individuals with disabilities | Survey / Questionnaire | Indicated factors that are associated with community participation | N/A |
| Vader et al., 2019^24^ | Explore barriers and facilitators for CMP | Interpretive description qualitative study | Adults with chronic pain | Interviews | Identified factors that rehabilitation should aim to focus on to promote CMP | N/A |
| Yin et al., 2020^25^ | Examines the social participation performance of wheelchair users using geolocational and lifestyle survey data | Observational study | Wheelchair users | Geolocational and lifestyle survey | Wheelchair users social participation performance is influenced by their geolocational travel patterns and other lifestyle factors | Duration of data collection (n=3 months) |
| Damen et al.  2020^30^ | Examining sports program impact on physical activity and fitness of children | Cohort Study | Manual wheelchair users | 6-Minute Push Test, physical assessment | 6-Minute Push Test shown as potential means of measuring community mobility | Study not focused on 6-Minute Push Test |
| Andrews et al., 2022^31^ | Examines the inter-rater reliability and reference value of Wheelchair propulsion test compare to performance between manual wheelchair types at different paces of wheelchair users | Cross-sectional descriptive study | Wheelchair users | Wheelchair propulsion test | Participants using their personal wheelchair has faster speed compared to those using a lightweight and ultralightweight wheelchair | Other wheelchair configuration were not considered |
| Van Der Westhuizen 2017^32^ | Examining relationships between physical fitness and community participation | Cohort Study | Manual wheelchair users | 6-Minute Push Test, Borg scale, RNLI | Provided evidence to support the 6-minute push test as well as for physical fitness as a means of measuring community mobility | N/A |
| Field et al., 2020^38^ | Evaluates the impact of wheeled mobility interventions using Wheelchair Outcome Measure for Young People (WhoM-YP) | Mixed method study | Children age 18 and younger who need wheeled mobility | Semi-structured interviews | Supported evidence in using WhoM-YP for measuring participation outcomes in daily life for young people | N/A |
| Filleke et al. 2019^39^ | Identifying GPS indicators of community mobility | Cohort Study | Older adults | GPS tracking data | Showed how effective GPS data can be for Community Mobility | Only utilized GPS Data |
| York et al. 2017^40^ | Examines GPS tracking of urban seniors | Observational Study | Older adults | Smartphone based GPS tracking | Found that older adults spends nearly 40 % of their time outside of their residential tracts | Local demographic |
| Zhu et al. 2020^41^ | Compares GPS sensor and self-reported community mobility measures | Comparative study | Older adults with Parkinson’s Disease | GPS sensor and LSA-life space assessment | GPS is a good replacement for dairies in assessing trip. Life space assessment may not reflect actual mobility | Limited applicability |
| Nanda et al. 2021^42^ | Examining community mobility after COVID-19 in Indonesia | Data Analysis | N/A | GPS Data | Gave a basis for GPS and accelerometer data to be useful in measuring community mobility and participation | No real sample size. |
| Sun et al.  2022^43^ | Examining community mobility and participation during the COVID-19 pandemic | Data analysis | COVID-19 isolated individuals | GPS data | Provided further support for using GPS data for measuring community mobility | No real sample size. |
| Bourassa et al. 2020^44^ | Validated actigraphy to measure physical activity intensity | Experimental Design | Manual wheelchair users | Actigraphy | Actigraphy, heart rate, and rating of perceived exertion covaried as physical activity intensity change | Physical activity was over a very short duration |
| Boissy et al.  2018^45^ | Examining mobility of at home older adults with wearable sensors | Cohort Study | Older adults | Self-report survey | Wearability and usability of wearable sensor devices shown to be a significant impactor | N/A |
| Seymour et al. 2019^46^ | Examining community rehabilitation in Uganda | Cohort Study | Wheelchair rehabilitation workers | Survey | Evidence for quality of rehabilitation equipment impacts community mobility and participation | Small scale study |
| Henschke et al., 2022^47^ | Assess the criterion validity between portable IMU sensors and optical motion for measuring kinematic parameters during shoulder movement | Validation Study | Those who need rehabilitation and have difficulty getting to a clinic | IMU, MoCap | Found that the IMU did not match the gold-standard well enough for clinical use. Suggested more innovation for IMU devices | Limited commercially available systems |
| Jang et al., 2023^48^ | Examined how out of clinic results differed to in-clinic results for the wheelchair skills test. | Mixed method study | Motorized mobility scooter | Wheelchair Skills Test (WST) | WST provides better reflection of everyday performance in out of clinic setting | N/A |
| **Factors Impacting Community Mobility and Participation** | | | | | | |
| Magasi et al. 2018^49^ | Examined participation outcomes based on mobility device quality | Cohort Study | Adults with SCI, traumatic brain injuries, and stroke | Device assessment, survey | Determined that the quality of mobility devices have an important role for rehabilitation results | N/A |
| Oldfrey et al., 2023^50^ | Investigates the practices of repair that exists for users of mobility assistive products in low resource setting | Qualitative study | Mobility assistive product users | Interviews, observation, surveys, | Community based repeair strateges should be incoportated into and supported by assistive technology provisions | N/A |
| Ferretti et al., 2022^51^ | Identifies and synthesize evidence on the effect of mobility and participation and quality of life of wheelchair users | Systematic Review | Adults who use wheelchair as their primary means of mobility | Literature review | Provided information on wheelchair mobility and its impact on community participation | N/A |
| Bazant et al., 2017^52^ | Provided information on the importance of wheelchair use services for wheelchair users | Cross sectional survey | Adult manual wheelchair users | Survey | Wheelchair services provided to wheelchair users is associated with an increase in activities of daily living performances (ADL) with wheelchair use | N/A |
| Nuri et al., 2022^53^ | Looks to understand users’ satisfaction with assistive device | Cross sectional survey | Assistive device users | Survey | The provision of assistive device needs a holistic approach by accommodating individuals’ need and the environment in which to function | N/A |
| Mattie et al., 2019^54^ | Examines the lived experience of wheelchair users with initiated seating adjustment and the impact of this feature in their daily lives | Exploratory study | Manual wheelchair users with user-initiated adjustability seating | Qualitative interviews | Adjustable seating can have significant impact on wheelchair users by increasing comfort, convenience, improved functionality, and participation | Small sample size (n=8) |
| Gowran et al., 2020^55^ | Examined how wheelchair and seating assistive technology provision impact on people’s live | Mixed method study | Wheelchair service users | Survey and interviews | Appropriate wheelchair and seating assistive technology provision is a basic human right and should be provided to wheeled mobility users’ | N/A |
| Hansen et al. 2020^56^ | Examination of potential barriers to mobility and participation in the community | Cohort Study | Manual wheelchair users | Survey | Provided solid outline on the impact of certain barriers on community mobility and participation | N/A |
| Prescott et al. 2020^57^ | Examining factors in the local community of the wheelchair user | Cohort Study | Mobility aided device users | GPS data | Identified community factors that could be modified to help the wheelchair user | N/A |
| Gharebaghi et al. 2021^58^ | Proposes a novel approach for computing user-specific route for people with motor disabilities | Experimental Study | Manual wheelchair users | Questionnaire, web-based GIS tool | Navigation system could provide a personalized routes that are tailored to their individual needs | N/A |
| Toro-Hernandez et al. 2020^59^ | Examined the factors that influences the use of community assets by people with disabilities | Qualitative Study | Individuals with disabilities | Interview, participatory mapping | There is a chain of contextual factors that limit access and use of assets stemming from the personal, interpersonal and community levels | Population type limitation |
| Koontz et al., 2020^60^ | Identifies facilitators and barriers to wheelchair transfers in the community | Survey | Wheeled mobility device users | Survey | Provides information on facilitators and barriers to wheelchair transfer in the community and also provides information on specific places in the community where increase transfer accessibility would increase participation in the community | N/A |
| Bezyak JL et al. 2017^61^ | Investigating barriers to community participation in public transportation for people with disabilities | Cohort Study | Individuals with disabilities | Online survey | Identified that attitudes, and lingering physical barriers can impact community mobility and participation | Nature of survey limited who was able to answer it |
| Grills et al. 2017^62^ | Estimated disability prevalence, and determine associated sociodemographic factors and compare access in the community between people with and without disability | Quantitative Survey | Individuals with disabilities | Survey | People with disabilities had significantly less access to services than those without. Reported barriers were lack of information, transport and physical inaccessibility. | Limited applicability |
| D’Souza et al., 2019^63^ | Determines the effect of low floor bus interior design and passenger load on wheeled mobility device user | Observational study | Wheeled mobility users | Simulated full scale low floor bus mockup simulating boarding and disembarking tasks | Highlights the importance of considering the design and layout of transport systems to improve accessibility and accommodations for wheeled mobility users | N/A |
| Holt et al., 2021^64^ | Evaluates the current barriers associated with gynecologic care as perceived by women who uses wheelchair | Qualitative study | Adult females who use wheelchair for mobility | Interview | Interventions are needed to improve accessibility car for women who use wheelchairs | Self-reported perception |
| Barbareschi et al., 2019^65^ | Investigates the personal needs, experiences and concerns of wheelchair users in relation to wheelchair transfers | Qualitative study | Wheelchair users and occupational therapists | Focus groups and interviews | Wheelchair transfers is difficult and transfer training is important to improve safety and reduce risk of overload injury | N/A |
| Serres-Lafontaine et al. 2023^66^ | Examines the impact of peer training and entrepreneurial skills training on social participation of individuals with spinal injury | Qualitative study | Wheelchair users | Qualitative photovoice | The training had a positive impact on the social participation of the individuals with SCI and the daily challenges faced were highlighted | Small sample size (n=10), lack of information on the level of SCI and subjective bias in the photo taken |
| Abou et al., 2022^67^ | Investigates the predictor of participation enfranchisement of individuals with SCI who use wheelchair | Secondary data analysis of a cross-sectional study | Wheelchair users | Survey | Mobility level, wheelchair skills, environmental barriers, symptoms of depression are factors that can influence participation of wheelchair users | Limited generalizability and applicability |
| Silveira et al., 2022^68^ | Examines association between individual wheelchair skills form the wheelchair skills test questionnaire and fitness | Secondary analysis of a cross-sectional study | Manual wheelchair users | Wheelchair skill test questionnaire and wheelchair exercise test | Wheelchair skills are significantly associated with fitness | N/A |
| Hansen et al., 2021^69^ | Examined whether the perception of physical activity barriers are associated with wheelchair users sociodemographic characteristics | Cross-sectional observational study | Manual wheelchair users | Survey | Manual wheelchair users who are obese or do not have education reported high physical activity barriers | Bias due to self-reported data |
| Sung et al., 2020^70^ | Examine if there are differences in CP and QOL for wheelchair users with and without the fear of falling | Cross-Sectional Study | Wheelchair user | Questionnaire | The presence of the fear of falling significantly lowered the CP of the participants | Causality isn’t clear. Data was self-reported |
| Borisoff et al. 2018^71^ | Examining season patterns of community mobility and participation | Cohort Study | Wheelchair users | GPS tracker, accelerometer, interviews | Snow and Ice significantly impact community mobility and participation | Data loss |
| Ripat et al., 2017^72^ | Explores the patterns of wheelchair users’ community participation across a year period to develop a understanding of the patterns and influences of wheelchair use in and participation of wheelchair users | Instrumental case study | Individuals who use wheelchair | GPS data logger and interview | Winter conditions created community participation challenges. Providing accessible transportation and accessible community environment are key in promoting participation | N/A |
| **How Rehabilitation can Improve Community Mobility and Participation** | | | | | | |
| Rice et al., 2019^73^ | Investigate the circumstances surrounding falls of MWU, the recovery process and how it influences CP | Mixed-Method Research Study | MWU | Interviews | Emphasized the complexity of how falls occur, and the need for assistance in recovery programs. | Self-Reported |
| Singh et al., 2020^74^ | Understanding factors that influence the risk of falling as perceived by wheelchair users with SCI | Qualitative study | Wheelchair users | Photo-elicitation interviews | Wheelchair users encounters different fall risk factors in their daily life | Limited transferability |
| Armstrong et al., 2018^75^ | Determines the feasibility of automatically restoring seated stability to manual wheelchair users with SCI with neural electric stimulation | Feasibility study | Manual wheelchair users | IMU, Vicon motion analysis, Implanted pulse generator (IPGs) | Application of neural stimulation during wheelchair propulsion after SCI injury can enhance recovery of upright siting after destabilizing events | Small sample size (n=4) |
| Chamran et al., 2023^76^ | Examine the central nervous system and how it controls human movement in order to improve FES rehabilitation therapy | Feasibility Study | MWU | Visual motion analysis | Gathered data considered a sufficient groundwork for future study for task-specific FES rehabilitation therapy | No participants had upper limb pain, injuries, or disorders. |
| Rice et al. 2017^77^ | Examined key evidenced based training interventions and educational protocols focused on preventing and managing upper limb pain and injury among full time manual wheelchair users. | Literature Review | Manual wheelchair users | N/A | Shows that fulltime manual wheelchair users are at high risk of developing upper limb injury and that Preventive methods are critical to prevent disruption of normal activities | N/A |
| Finley et al., 2017^78^ | Examines the relationship between the pectoralis minor muscle (PMm) length and extensibility of manual wheelchair users | Cross-sectional cohort study | Manual wheelchair users | Clinical measures  motion analysis | Individuals with greater than 15 years of wheelchair use had reduced PMm extensibility and reduced peak humerothoracic elevation that those with 5 years wheelchair use | Limited applicability |
| Remsik et al., 2018^79^ | Examines the rehabilitative efficacy of BCI intervention for upper extremity motor disability in stroke survivors | Randomized control trial | Stroke survivors | EEG based BCI intervention | BCI intervention improve Participants improve action research arm test scores as well as in their mobility and strength | Variability in the acquisition of study measure |
| Bockbrader 2019^80^ | Examines the use of BCI technology for individuals with SCI who have tetraparesis | Review | SCI individuals | Review BCI technology for SCI who have tetraparesis | BCI can restore upper limb sensorimotor function n individuals with tetraparesis, improving hand function and quality of life | N/A |
| Cervera et al., 2018^81^ | Evaluates the effectiveness of BCI for post stroke rehabilitation | Meta analysis | Post stroke survivors | Review of randomized controlled trial studies | BCI shows promise as a effective therapy for post stroke upper limb rehabilitation | N/A |
| Nojima et al., 2021^82^ | Evaluates the effect sizes of clinical studies investigating the use of BCI based rehabilitation interventions in restoring upper extremity function | A meta-analysis | Clinical trials that used BCI-based training for post-stroke patients. | Review | BCI-based training was determined to be superior to conventonal interventions during recovery of the upper linmbs | Results not conclusive due to a high risk for bias and heterogeneity |
| Nishimoto et al., 2018^83^ | Testing of a novel compact task-specific BCI system | Prospective before-after study | Severe chronic hemiparetic stroke patients | Fugl-Meyer upper-extremity motor and motor activity log-14 amount of use scores. Quebec User Evaluation of Satisfaction with assistive Technology | They concluded that their novel BCI system was feasible for use in real-world clinics | Study only examined a 10 day period of time, no analysis of long term impact |
| Shen et al., 2022^84^ | Analysis of a BCI active and passive rehabilitation training system | Cohot Study | Stroke patients | EEG | Showed the system’s potential to reach a high execution accuracy and evaluate participant’s participation in the rehabilitation. | N/A |
| Al-Taleb et al., 2019^85^ | Examines the feasibility of using a portable BCI for home based self managed treatment of central neuropathic pain in people with spinal cord injury | Usability study | SCI individuals | EEG, interview and questionnaires | BCI for neurofeedback training at home can effectively reduce central neuropathic pain in patients with spinal cord injury. | No explicit test for placebo effect |
| Mattia et al., 2020^86^ | To test the effectiveness of a “Promotoer” study meant to assess early hand motor function improvement from BCI-assisted MI training. | Longitudinal 2-arm randomized controlled superiority trial | Unilateral subacute stroke patients | Novel “Promotoer” study, Fugl-Meyer Assessment, Modified Ashworth Scale, Numeric Rating Scale for Pain, Action Research Arm Test, NIH Stroke Scale, and Manual Muscle Test | The trial provided evidence that BCI-based MI rehabilitation could promote long-lasting retention of early improvement in motor control for the hand. | N/A |
| Belkacem et al., 2020^87^ | Describes recent studies on cognitive decline and advancing BCI technologies to improve the health and wellness of older adults | Mini-Review | Older adults and elderly patients | Review | Described current trends and the innovation for BCI. | N/A |
| Carino-Escobar et al., 2019^88^ | Analyze stroke patients during a BCI intervention for upper limp rehab | Longitudinal study | Stroke patients | EEG | EEG with BCI intervention can help improve BCI rehab for upper limbs | Small sample size (n=9) |
| Camargo-Vargas et al., 2021^89^ | Reviews the development of BCI for the rehabilitation of upper and lower limbs | Systematic review | N/A | Review | EEG signals and user feedback in BCI for limb rehabilitation offers benefits like improved training, user motivation, and accessibility to users | N/A |
| Zhuang et al., 2020^90^ | Reviews non-invasive BCI for neural rehabilitation | Review | N/A | Review | BCI intervention using EEG has shown promise in restoring motor function and aiding in the recovery | N/A |
| Cajigas & Vedantam, 2021^91^ | Reviews recent progress in BCI and neurorehabilitation for SCI patients | Review | SCI patients | Review | Utilizing BCI and neurorehabilitation displays together has promise for superior outcomes compared to individual usage | N/A |
| Samejima et al., 2021^92^ | Demonstrate that BCI can be used to improve rat upper extremity functionality | Feasibility Study | SCI patients | Task performance | BCI stimulation restored control for the rat’s upper extremity | Study was performed on rats, not humans |
| Jovanovic et al., 2021^93^ | Assess the feasibility and clinical potential of using BCI-FEST for the rehabilitation of reaching and grasping in individuals with SCI | Feasibility study | SCI patients | BCI-FEST system | BCI triggered functional electrical stimulation therapy is safe and promising for rehabilitating after spinal cord injury. | Small sample size (n=5) |
| Jovanovic et al., 2020^94^ | Assessing the feasibility of BCI-triggered FEST to restore upper limb function | Case Study | One 57 year old man with a severe case of left hemiplegia due to a stroke 6 years prior | Functional Independence Measure, Action Research Arm Test, Toronto Rehabilitation Institute – Hand Function Test, and Fugi-Meyer Assessment Upper Extremity test | Improvement was demonstrated in the participant’s ability to perform day-to-day activities. | The test was conducted over the course of 14 weeks with no longer term efficacy evaluation |
| Jervis-Rademeyer et al., 2022^95^ | A Study conducted to understand the perspectives of therapists when it comes to using BCI-FEST and if it is feasible to use clinically on a large-scale | Qualitative Exploratory Study | Physical and occupational therapists who have used BCI-FEST | Interview with questions using the COM-B model of behavior change | Successfully identified limiting factors which may have an impact on clinical implementation which would need to be addressed | Only a total of 6 therapists were interviewed, low population sample size |
| Spicer et al., 2017^96^ | Development of a closed loop neurofeedback system called REINVENT to promote motor recovery in individuals with severe motor impairment after stroke | Feasibility Study | Stroke patients | EEG, EMG | Gathered sufficient evidence to further refine the REINVENT system for future research to establish feasibility and effectiveness | Very short experiment (1 hour) |
| Lakshminarayanan et al., 2023^97^ | Examining if using VR for action observation during kinesthetic motor imagery would be more effective than motor imagery | Feasibility Study | Healthy Patients | EEG, VR motion recording | They found that the VR-based action observation enhanced outcomes over motor imagery without the VR action observation | Lack of capacity for generalization to any scenario |
| Achanccaray et al., 2018^98^ | Applies BCI with VR to support upper limb rehabilitation therapy for post stroke patients | Experimental study | Healthy subjects | EEG and VR | BCI with VR feedback has positive effects in upper limb rehabilitation therapy | N/A |
| Lakshminarayanan et al., 2023^99^ | Investigate cortical activity and classification performance during tactile imagery to see if it is a viable alternative to motor imagery | Feasibility Study | Healthy subjects | Event-related desynchronization response and BCI classification performance | Tactile Imagery was shown to be a viable alternative to motor imagery, building support for its use alongside BCI in rehabilitation | Location of event-related potential component that could have interacted with the event-related desynchronization response |
| Sebastian-Romagosa et al., 2020^100^ | Examines the use of BCI therapy as a rehabilitation tool for to help patients with impaired upper extremity movement due to stroke | Feasibility Study | Stroke patients | EEG | BCI treatment is effective in promoting long lasting functional improvements in upper extremity in stroke survivors with severe, moderate and mild impairment | No control group was included |
| Vourvopoulos et al., 2019^101^ | Determine the efficacy of EEG-based BCI-VR systems | Clinical Case Report | A 60 year old man with chronic stroke | Functional MRI, Fugl-Meyer | Provided evidence and data showing the efficacy of BCI using MI for motor rehabilitation | N/A |
| Katri et al.  2021^102^ | Examination of home-based rehab after hospital discharge | Cohort Study | Hospital discharged older adults | Survey, accelerometer | Demonstrated the need to assess patients during hospitalization | Did not directly correlate rehabilitation and Community Mobility and Participation |
| Kirby et al., 2018^103^ | Examine the relationship between wheelchair skills scores and peak exercise capacity in community-dwelling manual wheelchair users with spinal cord injury | Cross-sectional study | Manual wheelchair users | Wheelchair skill test questionnaire | Significant relationships between wheelchair skills scores and peak exercise capacity in manual wheelchair users with spinal cord injury | Small number of female in the study |
| Sol et al., 2021^104^ | Examines the effects of wheelchair mobility skills (WMS) training and exercise training on physical activity, WMS, confidence in wheelchair mobility, and physical fitness. | Intervention study | Manual wheelchair users | WMS training, WMS confidence and physical fitness | Exercise and WMS training has positive long-term effects on physical activity (PA), WMS, confidence in wheelchair mobility, and (an)aerobic performance in youth using a manual wheelchair. | Training approach not tailored to the individual |
| Canori et al., 2023^105^ | Examines how social engagement facilitated by mobile technology can reduce lack of motivation as a barrier to physical activity in individuals with SCI | Pilot study | SCI individuals | Semi structured interviews | Social engagement and communication with peers who have similar functional mobility levels and life experiences can potentially improve motivation for PA in individuals with SCI. | Retention of the participants for the study |
| Lipert et al. 2021^106^ | Examines the effect of an active rehabilitation camp | Observational Study | Wheelchair users | Fitness tests | Regular activities during the camp improves the Physical performance | N/A |
| Livingstone & Paleg  2021^107^ | Rehabilitation impact with children | Literature Review | Young children | N/A | Gathered information indicating that rehabilitation could improve community participation. | Results based on other Article Review |
| Canori et al., 2023^108^ | Examines how social engagement facilitated by mobile technology can reduce lack of motivation as a barrier to physical activity in individuals with SCI | Pilot study | SCI individuals | Semi structured interviews | Social engagement and communication with peers who have similar functional mobility levels and life experiences can potentially improve motivation for PA in individuals with SCI. | Retention of the participants for the study |
| Willig et al. 2020^109^ | Examined evidence of the effects of community based upper-body exercise programs on the functional independence and quality of life | Systematic Review | Manual wheelchair users | Systematic review of articles | Resistance training improved functional independence | N/A |
| Pellichero et al. 2020^110^ | Exploring older wheelchair users and training program | Cohort Study | Manual wheelchair users | Interview | Found training programs to improve satisfaction and community participation | Only data from interview |
| Charlton et al., 2021^111^ | Explore outcomes on skill performance, confidence and frequency of wheelchair use from a wheelchair skills training program | Pilot Study | MWU with lower limb amputation | Wheelchair Skills Test Questionnaire-Verion 5.0, interviews | The Wheelchair Skills Training Program could improve wheelchair performance, confidence and frequency of use | N/A |
| Gauthier et al., 2018^112^ | Compare at-home self-managed high and moderate intensity training programs | Exploratory Randomized, Controlled, Open-label Trial | MWU | Questionnaire, Clinical Assessment | The programs were found to be both feasible and safe, though pain was observed during the high intensity training | Lack of long-term analysis |
| Divanoglou et al. 2019^113^ | Examining effects of community-based rehabilitation | Cohort Study | Wheelchair users | Survey, wheelchair skill test | Demonstrated the impact community-based rehabilitation programs can be. | Participants not entirely representative of target population |
| Madsen et al. 2020^114^ | Examined the link between community-based rehabilitation approaches in an outdoor setting | Systematic Review | Individuals with disabilities | Qualitative | Community based rehabilitation can promote participation and social inclusion opportunities for people with disabilities | N/A |
| Ouellet et al. 2022^115^ | Examined community-based peer led approach to wheelchair skills training | Pilot study | Wheelchair users | Sociodemographic and wheelchair use questionnaire | Community based peer led group wheelchair skills training program improve participation and wheelchair skills and use improve self-efficacy | Small sample size (n=8) Mostly male |
| Best et al., 2017^116^ | Evaluate the feasibility of a peer-led WheelSeeU program | Randomized Controlled Trial | MWU | Survey and general observation during trial | WheelSeeU resulted in perceived benefits from the program with no adverse effects | Methods of gathering data for analysis of the effectiveness of WheelSeeU were lacking in variety and effectiveness |
| Miller et al., 2019^117^ | Examines the effect of peer led wheelchair training on objective wheelchair skills in older adults | Randomized controlled trial | Older adults | Wheelchair skills test, wheelchair skills questionnaire, WheelCon-short form, wheelchair outcome measure, LSA, Late Life Function and Disability Index | Peer led wheelchair training program should not be dismissed as it may improve wheelchair use, self-efficacy, skill, and performance | N/A |
| Giesbrecht & Miller, 2019^118^ | Evaluate the effect of an mHealth wheelchair skills training program on rehabilitation for older MWU | Feasibility randomly controlled trial | Older MWU | Wheelchair skills test | The mHealth training program resulted in evident CP and wheelchair self-efficacy improvements | Lack of investigation into benefit retention over time |
| Leving et al., 2019^119^ | Investigating changes in wheelchair technique and mechanical efficiency during 5 weeks of active rehabilitation | Validation Study | SCI MWU | Exercise tests, activity monitor equipment | There was no improvement in propulsion technique, only an improvement in mechanical efficiency | Conducted over a very short span of 5 weeks, no long term effect analysis |
| Yang et al., 2021^120^ | Investigate the feasibility of a VR simulator for wheelchair propulsion performance simulation | Validation Study | SCI MWU | Questionnaire and interview | The VR simulator experience was reacted to positively by the participants, and accurately modeled wheelchair propulsion performance, demonstrating its potential for training MWUs | N/A |
